# Harmless sea snail parasite causes mass mortalities in numerous commercial scallop populations in the northern hemisphere

**DOI:** 10.1038/s41598-018-26158-1

**Published:** 2018-05-18

**Authors:** Árni Kristmundsson, Mark Andrew Freeman

**Affiliations:** 1 Institute for Experimental Pathology at Keldur, University of Iceland, Fish disease Laboratory, Keldnavegur 3, IS-112 Reykjavík, Iceland; 2 Ross University School of Veterinary Medicine, Basseterre, West Indies Saint Kitts and Nevis

## Abstract

Apicomplexans comprise a group of unicellular, often highly pathogenic, obligate parasites exploiting either one or two hosts to complete a full reproductive cycle. For decades, various scallop populations have suffered cyclical mass mortality events, several of which shown to be caused by apicomplexan infections. We report the first dual mollusc life cycle for an apicomplexan: a species highly pathogenic in various pectinid bivalve species, but apathogenic when infecting the common whelk as *Merocystis kathae*. The sympatric distribution of the common whelk and scallops in the North Atlantic makes transmission extremely effective, occurring via the gastrointestinal tract, by scavenging and predation in whelks and unselective filter feeding in scallops. Infective sporozoites from whelks utilize scallops´ haemocytes to reach muscular tissue, where asexual reproduction occurs. Phylogenetically, this apicomplexan is robustly placed within the Aggregatidae and its inclusion in analyses supports a common ancestry with other basal invertebrate apicomplexans. Scallops seem able to regulate low-level infections of *M. kathae* as they exist in normal populations while epizootics occur during high levels of exposure from locally infected whelks. A targeted removal of whelks from valuable scallop grounds would be advantageous to minimize the occurrence of *M. kathae* epizootics and prevent damaging economic losses.

## Introduction

Phylum Apicomplexa forms a group of unicellular spore forming parasites sharing a defining feature, the apical complex that comprises structural and secretory elements that facilitates interaction with the host cell^1^. They are obligate parasites which develop mostly inside the host cell, but degrees of epi- and extra-cellular development are known^2^. Their life cycle is either monoxenous (one host) or heteroxenous (two hosts) and includes many and diverse developmental forms representing asexual (merogony) and sexual reproduction (gamogony) with the formation of infective sporozoites (sporogony)^3^. Both monoxenous and heteroxenous species are described from marine molluscs. Despite reports of apicomplexans infecting molluscs dating back to the 19^th^ century^4^, they are poorly understood, compared to those infecting vertebrate hosts.

The pathogenicity of apicomplexans varies considerably between species and/or their hosts. As most species are obligate intracellular parasites they cause a level of pathology. Some are considered to have low pathogenicity while others are highly pathogenic, such as those causing malaria, toxoplasmosis and cryptosporidiosis in humans. Highly pathogenic mollusc-infecting apicomplexans are known, for example the one infecting scallops (herein referred to as scallop apicomplexan or SAP). SAP has been shown to be largely responsible for the total collapse of a population of Iceland scallop *Chlamys islandica* in Iceland^5^, and an unidentified apicomplexan negatively impacting oyster populations in New Zealand^6^. Furthermore, strong indications exist that SAP causes regular mass mortalities in a number of commercial scallop species inhabiting different geographic areas^5,7,8^. Kristmundsson *et al*.^7^, observed a great number of developmental forms, in infected scallops, suggesting that it could potentially have a monoxenous life cycle, but could not exclude the requirement of an obligate alternate host.

*Merocystis kathae*, is an apicomplexan infecting renal tissues of the common whelk, *Buccinum undatum*, in northern Europe. *M. kathae* originally described by Dakin^9^ more than 100 years ago, subsequently the life history of the parasite was described in more details^10,11^. Gamogony and sporogony was found to occur in the whelk but merogony was absent and thought to develop in an unknown alternate host. No molecular data exist for this genus but based on its morphological features it has been classified within the Family Aggregatidae^10,11^.

The aim of the present study was to assess whether SAP in scallops is conspecific to *M. kathae* in whelks, and to better understand the life cycle and transmission of the parasite between the two different mollusc hosts if conspecificity is confirmed. In addition, we place *M. kathae* in a phylogenetic context within the Phylum Apicomplexa.

## Results

### Microscopic examination

**Figure 1:**
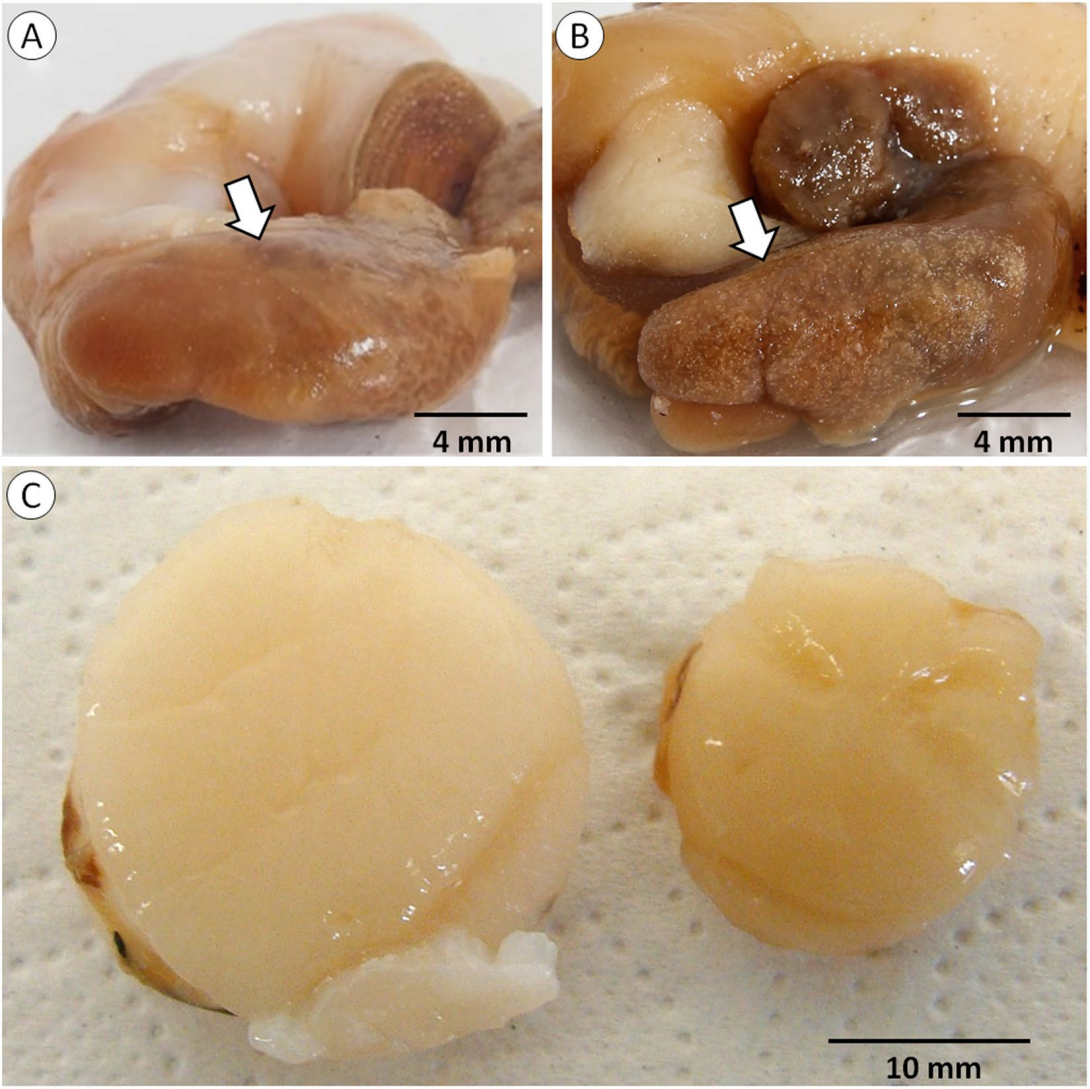
Macroscopic signs *Merocystis kathae* infection. (**A**) Normal, uninfected kidney of a common whelk. (**B**) Whelk kidney heavily infected with *M. kathae* characterized by numerous small white cysts visible to the naked eye. (**C**) Healthy and abnormal (right) scallop adductor muscles from equally sized shells. The muscles of scallops heavily infected with *M. kathae* are greatly reduced in size and have abnormal brown colouration.

Developmental stages of *Merocystis kathae* and the SAP were observed in all whelks and Iceland scallops examined, respectively. Gross clinical signs of infections were common, in whelks as small white cysts in the kidney (Fig. 1A and B) and in scallops as reduced abnormally brown-coloured adductor muscles (Fig. 1C). None of the king scallops, *Pecten maximus*, sampled from a scallop ranch in Scotland, where whelks are virtually absent, had clinical signs of disease and only few SAP life forms were detected in one of 20 scallops examined. Neither SAP nor *M. kathae* were observed in the other five species of gastropods and bivalves examined.

**Figure 2:**
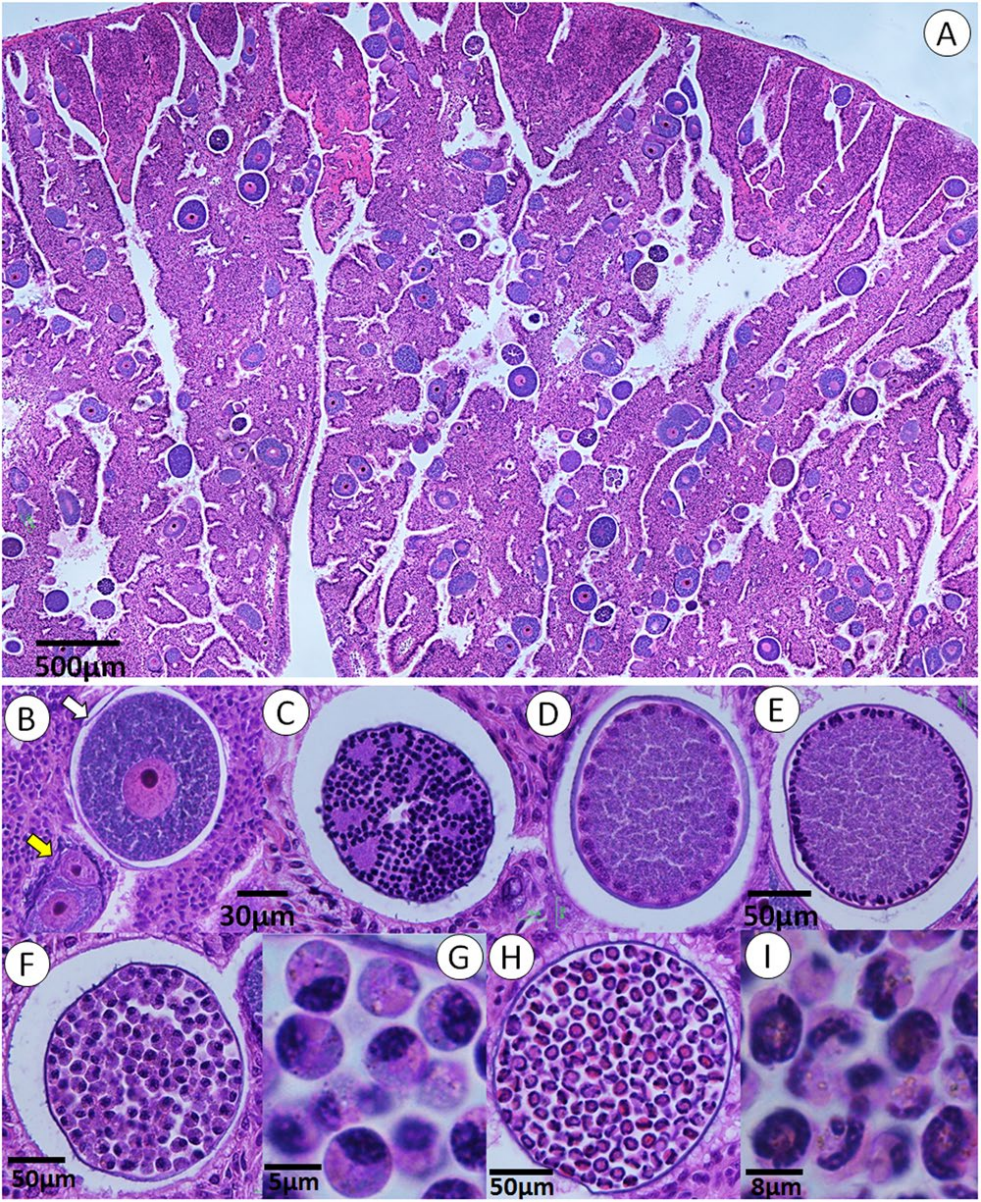
Histological sections of a whelk kidney infected with *Merocystis kathae*. (**A**) Kidney section of a common whelk heavily infected with *M. kathae* showing all gamogonic and sporogonic stages. (**B**–**I**) Higher magnification of the life forms; (**B**) mature macrogamont (white arrow) and two growing intracellular trophozoites inside renal cells (yellow arrow). (**C**) Mature microgamont with numerous microgametes. (**D** and **E**) Immature oocysts with peripherally located nuclei. (**F**) Premature oocyst filled with numerous sporoblasts. (**G**) Higher magnification of sporoblasts. (**H**) A mature oocyst filled with sporocysts, each containing two sporozoites. (**I**) Higher magnification of disporous sporocysts All sections are stained with H&E.

Most whelks were extensively infected (Fig. 2A) and all developmental stages, representing gamogony and sporogony, were observed in histological sections (Fig. 2B–I); the smallest forms detected were trophozoites around 10 µm, intracellular in renal cells.

**Figure 3:**
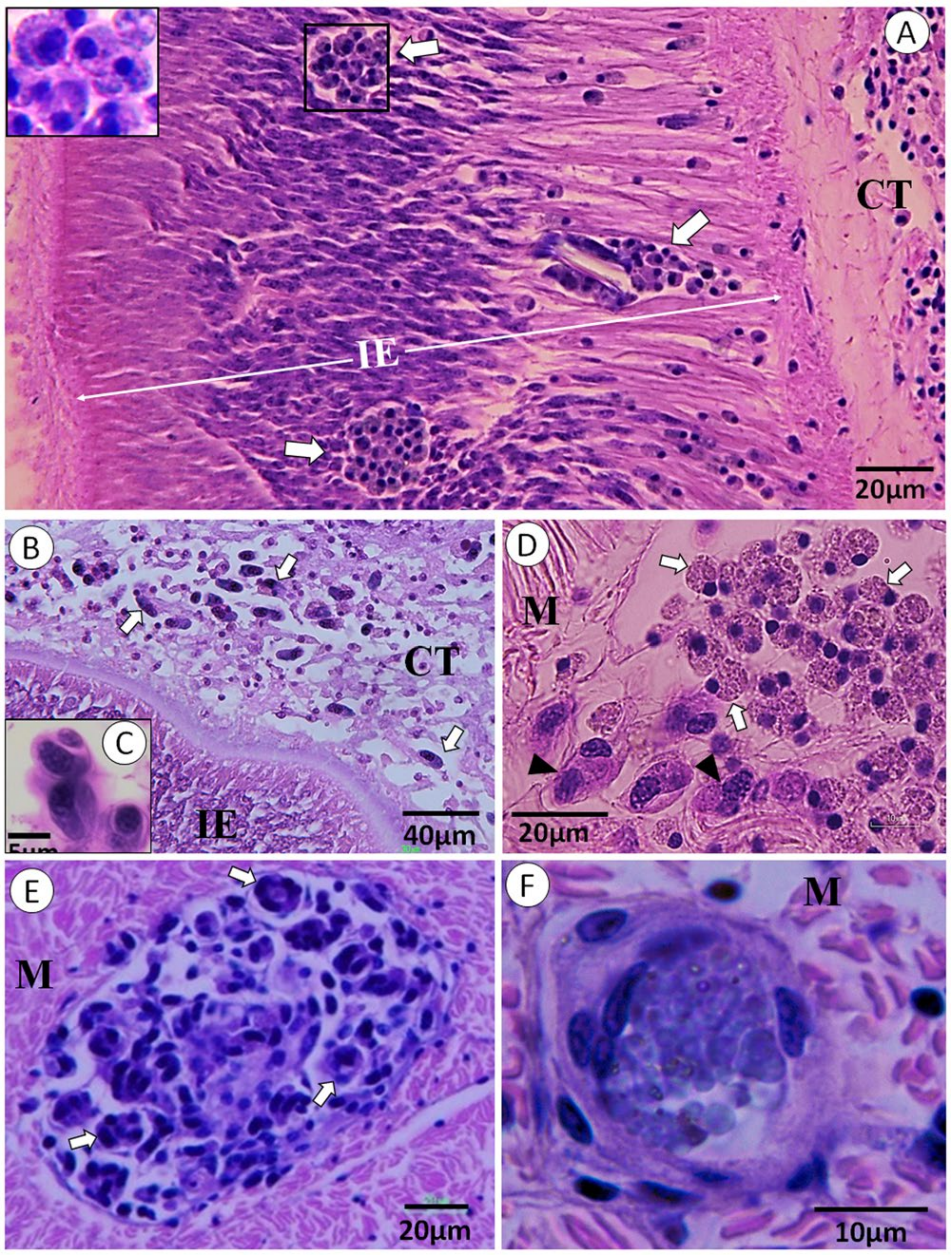
Histological sections of Iceland scallop infected with *Merocystis kathae*. (**A**) Histological section through the intestinal epithelium (IE) of Iceland scallop showing clusters of sporoblasts (arrows) of *M. kathae* entering the connective tissue (CT) adjacent to the epithelial lining. Inserted picture: Higher magnification of the sporoblasts. (**B**) Numerous sporozoites (arrows) which have entered the connective tissue between the intestinal epithelium (IE) and the digestive gland of Iceland scallop. (**C**) High magnification of two sporozoites inside haemocytes. (**D**) Cluster of sporoblasts (white arrows) and sporozoites (black arrowheads) in the adductor muscle of Iceland scallop. (**E**) Sporulated sporocysts (arrows) in the adductor muscle of Iceland scallop. (**F**) Initiation of merogony; young trophozoite developing inside an adductor muscle cell. All sections are stained with H&E.

**Figure 4:**
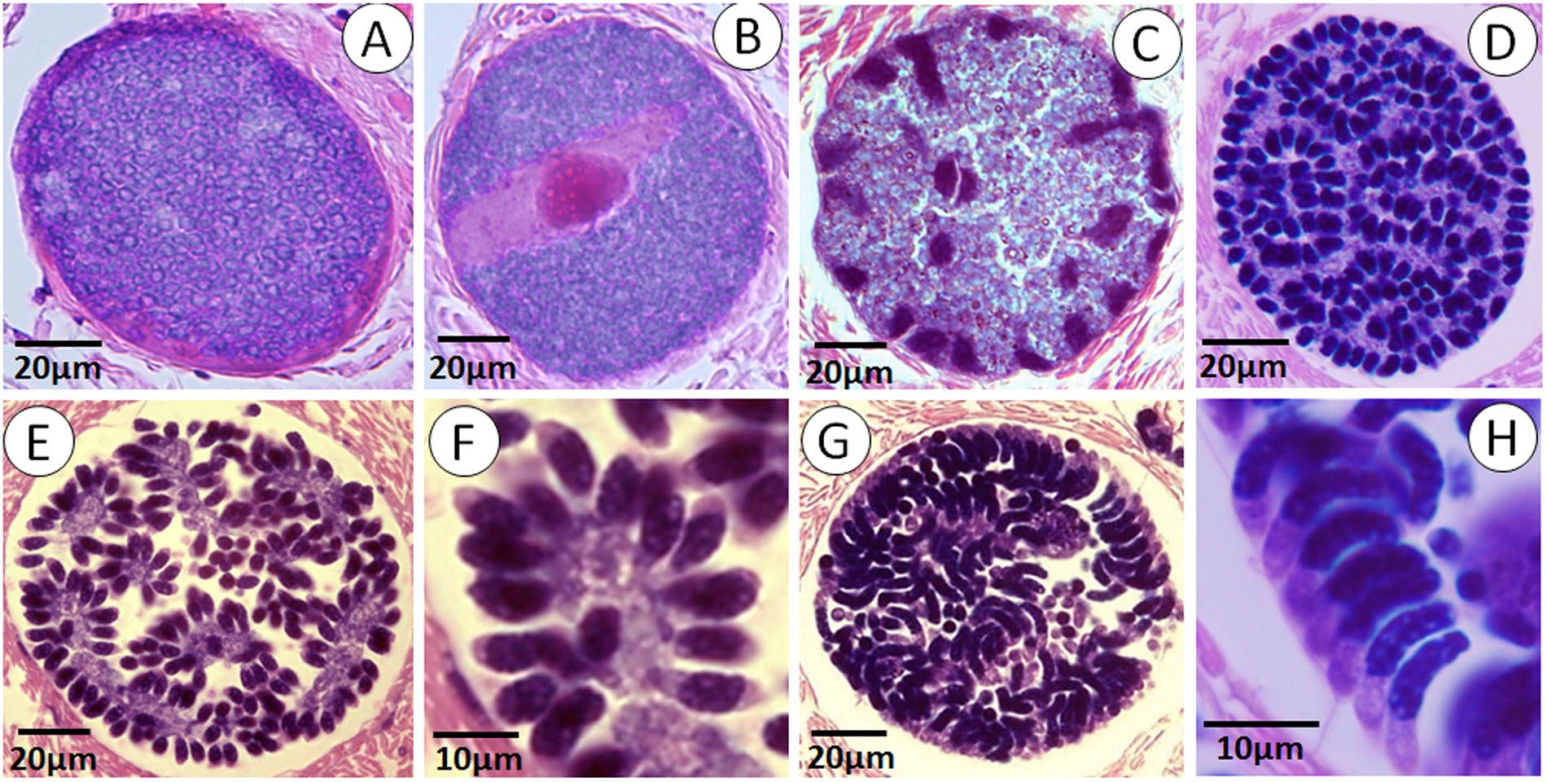
Merogonic stages of *Merocystis kathae* in the adductor muscle of Iceland scallop. (**A**) Premature meront. (**B**) A meront with a spindle-like apparatus indicating the initiation of nuclear cleavage. (**C** and **D**) Further development of a meronts characterized by further divisions. (**E**–**H**) Two different types of meronts representing two generation of merozoites. (**E**) Mature type I meront with numerous merozoites arranged in a rosette like fashion. (**F**) Higher magnification of merozoites from image (**E**). (**G**) Mature type II meront with numerous merozoites, more convex and slender in appearance and differently arranged compared to type I meronts. (**H**) Higher magnification of merozoites from image (**G**). All sections are stained with H&E.

In scallops, initial infections were sporoblasts and sporozoites in the gastrointestinal epithelium and later in the adjacent connective tissues associated with the digestive gland and gonads (Fig. 3A and B). These forms appear identical to sporoblasts and sporozoites which develop in the whelk (Figs 2F–I and 3D). The sporoblasts commonly sporulate in the connective tissues, resulting in a sporocyst with two sporozoites. At these sites, the sporozoites are often seen inside haemocytes (Fig. 3C). In addition, sporoblasts are occasionally seen in the adductor muscle (Fig. 3D). The target organ of the infective sporozoites, which develop in whelks and to some extent in scallops, is muscular tissue, especially the adductor muscle (Fig 3E). The sporozoites actively invade muscle cells which become hypertrophied and eventually rupture. It appears that only a fraction of the sporozoites develop further in the scallops, i.e. enter the merogonic phase. The first indication of merogony is the presence of 15–20 µm trophozoites inside muscle cells (Fig. 3F). They significantly increase in size and develop into early meronts (Fig. 4A). Subsequent development involves recurrent nuclear cleavage giving rise to multinucleated premature meronts with regularly arranged nuclei (Fig. 4B–D) which eventually become mature meronts containing numerous merozoites (Fig. 4E–H). Two generation of merozoites are present (Fig. 4F and H) originating from two types of meronts (Fig. 4E and G) with morphologically different merozoites; type I being shorter and with both ends somewhat pointed (Fig. 4F) while type II is convex, more slender and sausage-shaped (Fig. 4H).

SAP causes severe histopathological changes in the Iceland scallop which was comprehensively described by Kristmundsson *et al*.^5^. The histopathology of *M. kathae* in the whelks is minor, even in those with extensive infections. Regardless of infection status the whelks appear to be in good condition. As an intracellular parasite it causes some focal pathological changes in affected cells, i.e. the renal epithelial cells, which increase in size as the parasite grows and hence projects into the renal cavity or the underlying connective tissue. The host cell retains its position in the renal epithelium with no signs of penetration of the parasite into other host cells.

### PCR, DNA sequencing and *in situ* hybridization (ISH)

All Iceland scallops and whelks tested positive using a diagnostic PCR, initially developed for the SAP^5^. Furthermore, DNA sequencing showed that the SSU rDNA of *Merocystis kathae* and SAP was 100% identical.

**Figure 5:**
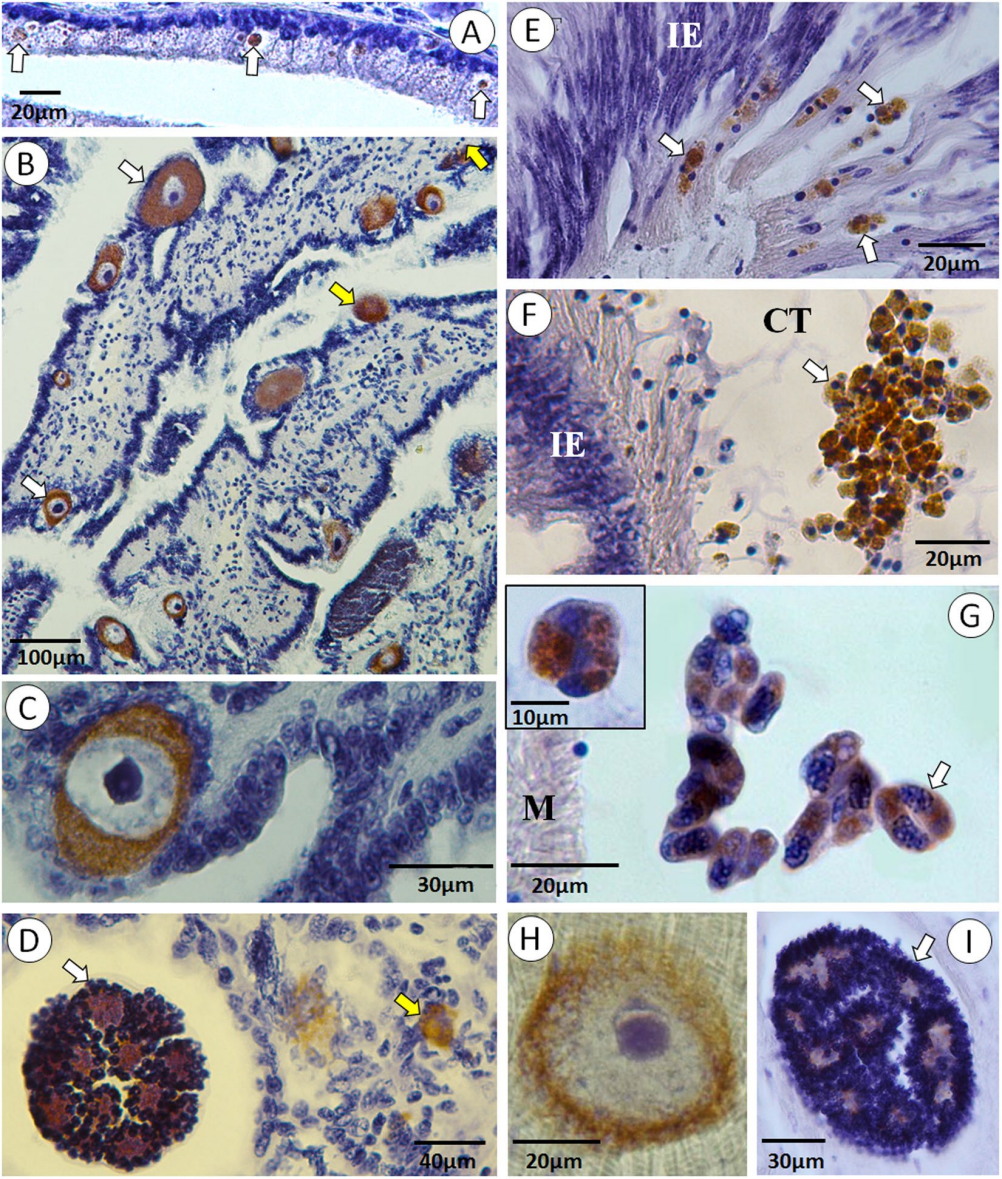
*In situ* hybridization of *Merocystis kathae* from whelks and scallops. Developmental stages in the common whelk (**A**–**D**) and the Iceland scallop (**E**–**I**). (**A**) A positive reaction to *M. kathae* in the intestinal epithelium of a whelk (white arrows). (**B**) Kidney section showing numerous developing gamonts (white arrows) and trophozoites (yellow arrows). (**C**) Higher magnification of a mature macrogamont. (**D**) Mature microgamont (white arrow) and a trophozoite (yellow arrow) inside renal cell. (**E**) A section through the intestinal epithelium (IE) of an Iceland scallop showing *M. kathae* sporoblasts (white arrows) entering the host. (**F**) A cluster of sporoblasts in the connective tissue (CT) adjacent to the intestinal epithelium (IE) of an Iceland scallop. (**G**) A group of sporozoites in the scallop’s adductor muscle (**M**). Insert shows a sporocyst with two sporozoites. (**H**) Developing meront in muscular tissue of a scallop with a large nucleus and a prominent nucleolus. (**I**) A mature meront with numerous merozoites in the adductor muscle of Iceland scallop.

ISH further confirmed the conspecificity of *Merocystis kathae* and SAP as all the developmental forms detected in both the whelks and the scallops gave strong positive reactions to the specific probes (Fig. 5A–I). Furthermore, small (5–6 µm) intracellular forms in the intestinal tract of the whelks showed a positive reaction for the parasite DNA in ISH (Fig. 5A), indicating that the transmission of the parasite, from scallops to whelk, occurs via the gastrointestinal tract.

### Proposed life cycle of  *Merocystis kathae*

**Figure 6:**
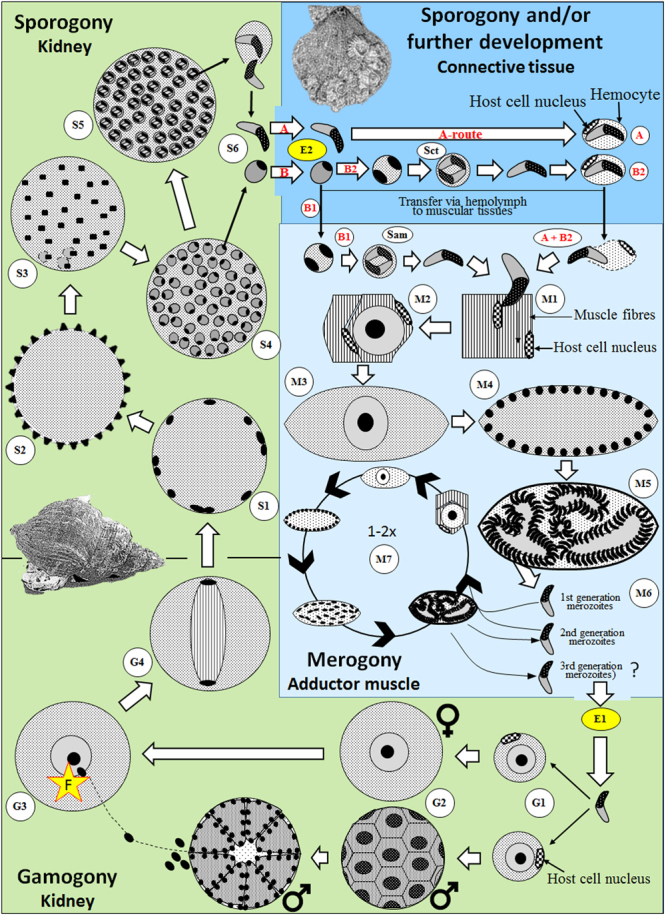
Schematic drawing of the proposed life cycle of *Merocystis kathae*. Merozoites invade the whelk through the intestinal tract (E1) and migrate to the kidney where they infect renal cells and gamogony **(G)** starts (G1). Some develop into macrogamonts (♀) while others become microgamonts (♂) (G2). The gamonts mature, eventually leading to fertilization (F) (G3) and the formation of a zygote which starts nuclear division (G4) initiating the sporogony process **(S)**. Subsequent recurrent nuclear cleavage occurs at the periphery of the zygote (S1) resulting in a cyst with regularly arranged nuclei at the periphery (S2). With further development the nuclei migrate into the cyst and start forming uninucleate sporoblasts, each containing cytoplasm (S3 and S4). The sporoblasts divide to form an oocyst with numerous sporocysts, each containing two sporozoites (S5). Sporogonic stages are released from the common whelk, either in the form of mature sporozoites (route A) or sporoblasts (route B), and enter the Iceland scallop via the gastrointestinal tract to invade the host via the intestinal epithelium (E2) and into adjacent connective tissues. For route A, the sporozoites are transmitted via haemolymph, commonly inside haemocytes, to muscular tissues. For route B, the sporoblasts are either transmitted directly to muscular tissues (Sam) where they sporulate (B1) or they sporulate in the connective tissues (Sct) surrounding the gastrointestinal tract prior to transportation to muscular tissues (B2). The merogonic phase (M) starts when the sporozoites invade muscle cells (M1). The muscle cells become hypertrophied as the pre-meront increases in size (M2), eventually leading to rupture of the muscle cell (M3). Further development of the meront is characterized by recurrent nuclei cleavage resulting in a multi-nucleated cyst (M4) which forms into a mature meront containing numerous merozoites (M5). Free merozoites, which are released from the meronts (M6), then infect new muscle cells starting a new merogonic cycle in the scallop’s muscle. After the formation of 2–3 generations of merozoites (M7), the last generation merozoites infect the whelk (E1) where the gamogonic phase starts again (G1).

The life cycle of *Merocystis kathae* is proposed in Fig. 6. In brief, whelks acquire infections via the gastrointestinal tract. The apicomplexan merozoites migrate to the kidney where gamogony is initiated by active invasion of the parasite into renal cells. Micro- and macrogamonts develop, leading to fertilization and the formation of a zygote. Subsequently, the sporogonic phase is initiated when the zygote nucleus divides followed by a series of further nuclear divisions, the end product being an oocyst with numerous sporocysts, each containing two sporozoites. In scallops, the transmission of *M. kathae* occurs via the gastrointestinal route; i.e. an active invasion, by either mature sporozoites or immature ones (sporoblasts), into connective tissues through the gastrointestinal epithelium. The final location for the parasites are muscular tissues where merogony takes place. Two generations of merozoites are produced; the latter one being infective to whelks.

### Phylogeny of *Merocystis kathae*

**Figure 7:**
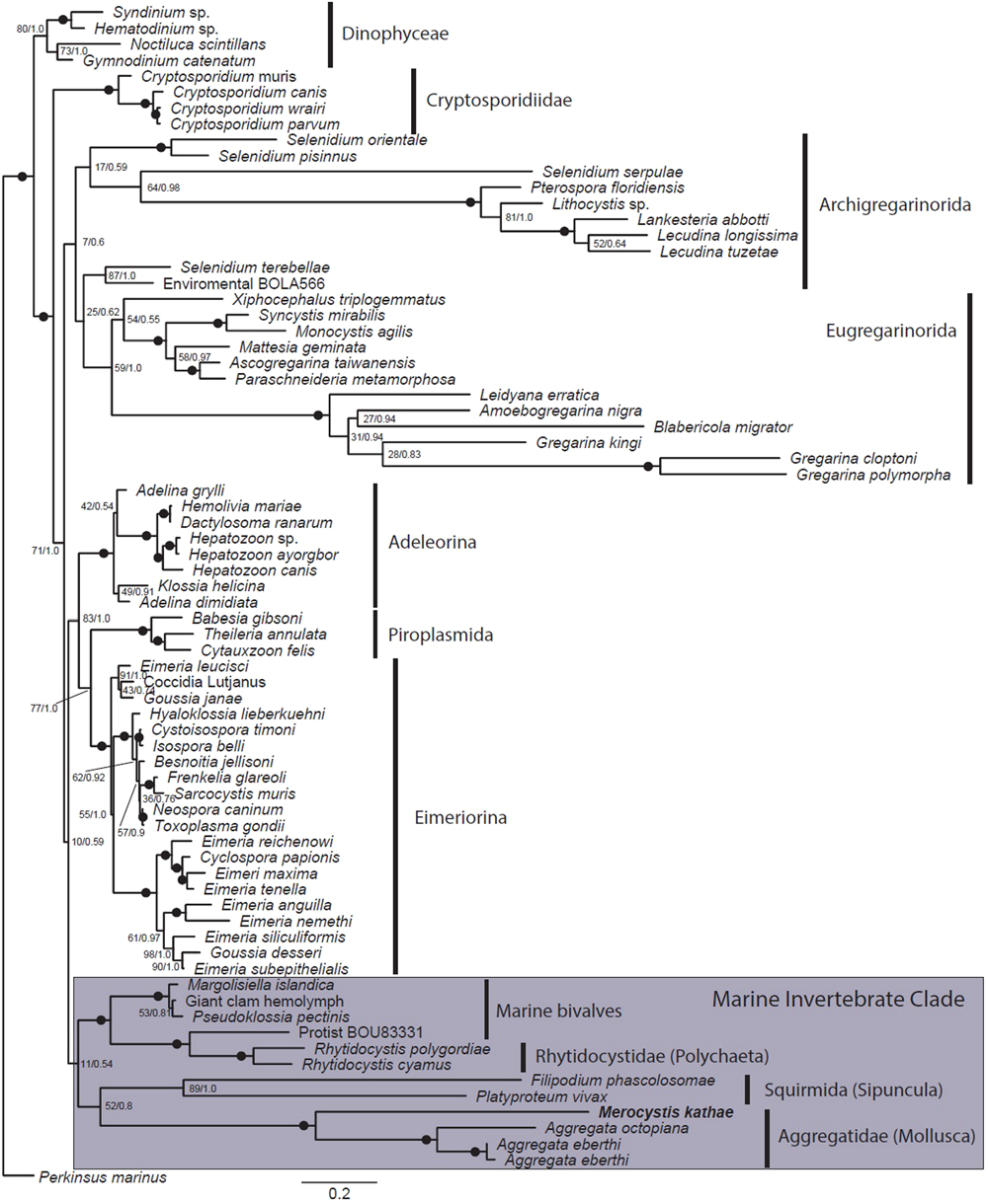
Phylogenetic tree. Maximum Likelihood (ML) topology of alveolate taxa focused on apicomplexans and rooted to *Perkinsus marinus*. The phylogenetic tree was inferred using the GTR + G + I model of substitution on an alignment of 74 small subunit (SSU) rDNA sequences and 1,916 sites. Numbers at the branches denote ML bootstrap percentages and Bayesian posterior probabilities; black dots on branches denote bootstrap and Bayesian posterior probabilities support of 95% and higher. *Merocystis* is fully supported in a clade with taxa from the genus *Aggregata*, in the marine invertebrate clade.

SSU rDNA sequences obtained from infected scallops have been confirmed as identical to DNA sequences obtained from whelks infected with *Merocystis kathae*. Phylogenetic analyses consistently and robustly place *M. kathae* in a clade with other members of the Aggregatidae (Fig. 7). This aggregatid clade is weakly, but consistently, associated with a sister clade containing *Filipodium phascolosomae* and *Platyproteum vivax* (Archigregarinorida (Squirmida)) from sipunculids. Other sequenced scallop-infecting apicomplexans, *Pseudoklossia pectinis* and *Margolisiella islandica* do not form part of this clade, but form a group of apicomplexans that infect marine bivalves and polychaetes (Rhytidocystidae). This entire group forms a weakly-supported clade of apicomplexans that are found in marine invertebrates (Fig. 7).

## Discussion

*Merocystis kathae* is the first apicomplexan parasite known to require two mollusc hosts to complete its life cycle, the common whelk and pectinid bivalves (scallops). It is the type species for the genus and was originally described by Dakin in 1911^9^, from the renal organ of common whelks from Port-Erin, Isle of Man.

Dakin^9^ placed *M. kathae* within the Family Polysporocystidae but later Foulon^10^, who described all life stages of the parasite in whelks in details, transferred it to the Family Aggregatidae and suggested it should be moved to the genus *Aggregata*. Patten^11^, who studied the seasonal life cycle of the parasite supported the suggestion that *M. kathae* should be placed within the Aggregatidae, but believed it was different enough from *Aggregata* species to retain the genus *Merocystis*. In these original descriptions^9–11^, only gamogonic and sporogonic stages were observed and it was hypothesised that merogonic stages must exist in an unknown host. Patten^11^ pointed out the similarity of the life histories of *M. kathae* and *Aggregata* species and the possibility that the missing host could be a crustacean, as was already known for *Aggregata eberthi*^12,13^.

Species within the Family Aggregatidae, are defined as parasites infecting marine invertebrates, especially molluscs and annelids, having all conventional apicomplexan life stages, mostly heteroxenous with merogony in one host, gamogony and sporogony in another and typically having oocysts with numerous sporocysts^14^. Therefore, with regard to morphology and life cycle, *M. kathae* conforms well to the family description. Currently the most speciose genus of this family is *Aggregata* with more than 20 nominal species, all of which are found in cephalopods, where the gamogonic and sporogonic stages are found^15^. In cases where the life cycle has been resolved, the merogonic stages have been observed in crustaceans, from which the cephalopods acquire infections by consuming infected individuals^12,14^. Therefore, the definitive hosts for both *Aggregata* spp. and *M. kathae* are molluscs. However, merogony in *M. kathae* occurs in pectinid bivalves not crustaceans as in *Aggregata* spp. Not much is known for species of other genera presently classified within the family Aggregatidae, except that the sexual development occurs in various marine invertebrates while the asexual ones are presumed to occur in unknown hosts^16,17^. The genera *Pseudoklossia* (Aggregatidae) and *Margolisiella* (Eimeriidae) are the exception for this including a number of known species, some recently described^18^. The genus *Margolisiella* was created to accommodate *Pseudoklossia* species known to be monoxenous and hence placed in the Family Eimeriidae. The remaining and expected heteroxenous species were left within the genus^19^ and retained in the Family Aggregatidae. The current classification of the genus *Pseudoklossia* within this family is questionable as many, and possibly all species, are monoxenous, and our current phylogenetic analyses support this theory placing the genera *Pseudoklossia* with *Margolisiella* away from the true Aggregatidae.

The phylogenetic placement of *M. kathae* in a clade with *Aggregata* spp. is fully resolved and creates a monophyletic grouping for the family, assuming *Pseudoklossia* species are not aggregatids. This is further supported by comparable studies of life cycles and developmental stages in other members of the family. However, support for the larger clade of apicomplexans infecting marine invertebrates (Marine Invertebrate Clade) is poor, yet consistent. These poor support values increase significantly, if the analyses are run without the Archigregarinorida (Squirmida) taxa (data not shown). However, when included, these taxa are consistently grouped within the Marine Invertebrate Clade as a sister to the Aggregatidae. *Filipodium phascolosomae* and *Platyproteum vivax* were both considered to be marine archigregarines in the family Selenidiidae^20^, but have been recently reclassified in a new taxonomic order, the Squirmida^21^. In previous phylogenetic analyses^20^ these taxa were found to be related to the archigregarines, whilst later ones^21^ found no clear phylogenetic placement within the Apicomplexa, rather a weak association with the Dinozoa.

In the current study, we find a consistent phylogenetic placement of *F. phascolosomae* and *P. vivax* in the marine invertebrate clade, with no reliable association with the Dinophyceae or the perkinsids as suggested by Cavalier-Smith^21^. Currently little sequence data are available for this diverse group of apicomplexans infecting marine invertebrates and their relationship with other basal apicomplexans such as the marine archigregarines, will without doubt become more apparent when more data is available for this very under-sampled group of apicomplexans.

An apicomplexan life cycle involving two different mollusc species is previously unreported, which may reflect the limited examination of invertebrate apicomplexans rather than it being an unusual occurrence. The present knowledge of the geographical distribution of *M. kathae* in the definitive host is also poorly understood, mostly due to limited research on parasites of the common whelk and related species. To date, this parasite has been reported from the Irish Sea^9–11^, the Belgian part of the North Sea^22^, in Øresund and Gullmarfjord in Danish- and Swedish waters^23^, and now Iceland in the present study. This novel discovery of the conspecificity of *M. kathae* and the SAP has extended the known distribution of this parasite. The presence of *M. kathae* in the intermediate scallop host, was first reported in 2011, from three different scallop species, *Chlamys islandica*, *Aequipecten opercularis* (queen scallop) and *Pecten maximus* (king scallop), in Icelandic-, Faroese- and UK waters, respectively^7^. Recently, it was also reported from *Placopecten magellanicus* (sea scallop), on the western side of the Atlantic; off the east coast of the USA and Canada^8^. All the species found infected with *M. kathae* were collected within the known distribution of the common whelk. In fact the distribution of these bivalve species is almost identical to that of the common whelk (Supplementary Information Fig. S1). The presence of whelks in more than 75% of tows during scallop fisheries also confirms the coexistence of these two mollusc species^24^.

Our present knowledge of host specificity of *M. kathae* is limited. However, the fact that it was not found in other non-pectinid bivalve species examined from same sites as infected whelks, suggests that it is not a generalist parasite infecting many unrelated bivalve species. It however seems quite possible that other species of the genus *Buccinum*, which includes close to 70 species^25^, could serve as hosts. The host range with regard to intermediate hosts, seems limited to pectinid bivalves. The two *Aggregata* species with known life cycles, seem more specific with respect to their cephalopod definitive hosts than their intermediate hosts which include various crustacean species of different families^26,27^. Therefore, it might be reasonable to think the same might be the case for *M. kathae*, being perhaps limited to certain whelk species but numerous pectinid hosts.

The transmission of *M. kathae* occurs via the gastrointestinal route in both the definitive and intermediate hosts. As unselective filter feeders, scallops consume a range of particle sizes from their surroundings by movement of ciliated cells in the gills; particles become entangled in mucus and are subsequently transferred along rejection tracts to the mouth palps, where they enter the digestive tract^28^.

*M. kathae* follows a seasonal pattern in whelks^11^, with the earliest developmental stages appearing between March and June while the first mature spores form in January and become increasingly common up to May. Consequently, the scallops are most extensively exposed to infective spores in late winter and spring. During an epizootic in the Iceland scallop population in Bay Breidafjördur in Iceland in the 2000s, the scallops caught in the spring were significantly more infected with SAP and associated macroscopic signs, than those caught in autumn^5^. The energy demanding maturation process, being close to spawning at that time of year, was suggested to make the scallops more vulnerable to infections. Although a plausible factor of influence, the extensive influx of infective sporozoites into their surroundings during this time must also play a major role. Whelks are known to be predatory, with scallops forming a regular part of their diet; they are also scavengers, feeding on moribund and dead animals^24,29^. Thus, during such mass mortality events^5^, the availability of dead or moribund scallops would be plentiful, resulting in whelks intensifying their infection. Subsequently, substantial amounts of infective sporozoites are released into the surroundings which infect the remaining naive filter feeding scallops. The very high prevalence of *M. kathae* in both whelks and Iceland scallops, with many heavily infected, reflects this situation.

All available studies indicate that *M. kathae* does not negatively impact the whelk definitive host^9–11^, as they are in good condition and the histopathological effect of the parasite is minor, even in extreme infections the, limited to hypertrophy of infected cells. However, there is no doubt that *M. kathae* is a serious pathogen of scallops, playing a major role in the sudden 90% decline in populations of Iceland scallop in Breidafjördur Iceland, severely affecting the queen scallop population around the Faroe Islands^5,7^ and a suspected cause of other mass mortality of sea scallops on the East coast of N-America associated with a condition called “grey meat”^8^. In addition, it has likely played a role in other unresolved mass mortality events and abnormal condition in various other Iceland scallop population, e.g. other scallop populations in Icelandic water^5,30–32^, but also in Norway^33^, Jan Mayen, Svalbard^34,35^, Greenland^36^, Russian Barents Sea^37^ and North shore of Quebec eastern Canada^38^. Furthermore, the abnormal condition of adductor muscles, similar to the one caused by *M. kathae*, observed in the weathervane scallop, *Patinopecten caurinus* in the Alaska Bay NE-Pacific Ocean^39,40^. This condition is associated with an apicomplexan infection, which according to Inglis *et al*.^8^ is likely to be *M. kathae*.

Although *M. kathae* has been shown to severely affect scallops, it appears that it only occurs when infections reach a high intensity, as low-level infections exist in scallop populations under normal conditions. After an almost complete collapse, the scallop population in Iceland has been slowly recovering and macroscopic disease signs are rarely detected and muscle and gonad condition appear normal. However, low-level infections still remain in high prevalence in the stock^5^. Similarly, highly prevalent but low level infections of *M. kathae* were observed in both king and queen scallops from UK waters but no abnormal clinical signs were reported^7^. This might suggest that the scallops’ immune system can to some extent suppress light infections.

Historical data on stock indices and mass mortality events in scallop populations are generally poorly documented and in most the aforementioned cases, the causes cannot be verified. However, considering the wide distribution of *M. kathae* and the fact that almost all these events occurred within the known distribution of the common whelk (Supplementary Information Fig. S1), it seems plausible that it was a major factor influencing these events. The only exception is the apicomplexan found in the weathervane scallop in Alaskan waters associated with the “weak meat” phenomenon^39,40^. Whether that apicomplexan is *M. kathae* is presently unknown but the definitive host would then most likely be a different whelk species, such as the sinuous whelk *Buccinum plectrum*. Although scarce, some reports exist from scientists and fishermen showing that mass mortality events in some scallop populations are cyclical. That is the case for sea scallops on the East side of North America associated where mass mortality events associated with a condition termed “grey meat” have periodically occurred since 1936^8^. Furthermore, some indications of such periodic events exist in Icelandic waters^5^. As *M. kathae* has been reported in relation with such events in both these scallop populations, it seems possible that epizootics occur regularly as a consequence of a host-parasite relationship, a phenomenon widely recognized in nature and in many ways comparable to prey – predator systems. In both cases, severe fluctuations occur in the population size of both groups^41^.

The main commercial fishing grounds for both scallops and whelks in Iceland is Breidafjördur. Until the collapse of the scallop population, commercial fisheries of that species had been reliably conducted since 1969. Whelk fisheries have a much shorter history, starting with some experimental collections in 1996 and have been quite intermittent to the present with no harvesting during some years^42^. Conversely, whelk fisheries are among the most important shellfish fisheries in the UK, dating back to the early 1900s^43^. As low-level infections do not appear to have a negative impact on the scallops, it should be possible to lower the infectious load with reasonable fisheries from both the whelk and scallop stocks. This would minimize the chance of epizootics caused be *M. kathae*, and create an optimal host - parasite equilibrium. The present study showed that infections were almost absent in king scallops from a “whelk free” area. Furthermore, only light infections were reported in both king and queen scallops from other UK locations, collected in 2007^7^. The extensive fisheries for whelks in the UK might help to explain this phenomenon.

## Materials and Methods

### Research material

In order to maximize the likelihood of detecting all life stages present in both host species, whelks and scallops were collected at different times during the years 2006–2016. Forty individuals of each species from different sampling times, showing gross signs of apicomplexan infections, were selected for examination. Samples were acquired by dredging in Bay Breidafjördur off the Wwest coast of Iceland from research expeditions performed by the Marine Research Institute in Iceland or from commercial whelk fisheries companies. All whelks and scallops were brought live to the laboratory and immediately dissected and examined for macroscopic/clinical signs of infections. Subsequently, the presence of *Merocystis kathae* and SAP was confirmed the by microscopic examination, for both whelks and scallops. All infected tissues were then subjected to conventional histological examination, while *in situ* hybridization (ISH) and molecular analyses were applied to the 10 most heavily infected individuals from each host species.

To check whether *M. kathae* and SAP could be generalist parasites, i.e. infecting a wide range of different host, five other mollusc species were collected from areas known to be endemic for SAP in scallops and *Merocystis kathae* in whelks, were examined for the presence of the apicomplexans. These were: rejected neptunes (*Neptunea despecta*, Gastropoda) (N = 10), dog whelks (*Nucella lapillus*, Gastropoda) (N = 10), blue mussels (*Mytilus edulis*; Bivalvia) (N = 30), northern horse mussels (*Modiolus modiolus*; Bivalvia) (N = 10) and the ocean quahogs (*Arctica islandica*; Bivalvia) (N = 30).

To further support the role of whelks in the life cycle of SAP*/M. kathae*, king scallops (*Pecten maximus*), were collected from a scallop ranching facility on the North West coast of Scotland (N = 20). Whelks are rare in the vicinity of the scallop beds and the farmers regularly remove the few individuals present during observational scuba diving inspections. All major organs of the scallops were subjected to a conventional histological examination. Furthermore, to assist with the phylogenetic placement of aggregatids from bivalves, samples of *Pseudoklossia pectinis* were taken from these scallops, the parasite identified from the kidney microscopically and samples taken for molecular analyses.

### Molecular work

Kidney samples from 10 whelks, 4 king scallops, and adductor muscle samples from 10 Iceland scallops were sampled directly into a lysis buffer for genomic DNA extraction using a GeneMATRIX kit (EURx Poland) following the tissue protocol. Apicomplexan small subunit ribosomal DNA (SSU rDNA) was amplified from the parasite using the primers and PCR conditions as previously described by Kristmundsson *et al*.^5^. In addition, the primer pairs 18e/SC2–1370r 5’ tccttcatatgtctggcactag 3′ and SFC-1120f 5′gaacgaaagttrggggmtcg3′/18 gM^44^ were used following the same PCR protocol. PCR conditions were as previously described but used an annealing temperature of 64 °C with an extension time of 30 s. PCR bands of the expected sizes were recovered from the PCR products using a GeneMATRIX PCR extraction kit (EURx Poland). All PCR reactions were performed in triplicate. Sequencing reactions were performed using BigDyeTM Terminator Cycle Sequencing chemistry utilising the same oligonucleotide primers that were used for the original PCRs. DNA sequencing was performed in both forward and reverse directions for all PCR products and nucleotide BLAST searches performed for each sequence to confirm an apicomplexan origin. The contiguous sequences were obtained manually using CLUSTAL_X and BioEdit^45^.

### Histology and *in situ* hybridization

Tissue samples from all Iceland and king scallops (N = 60) and whelks (N = 40) were fixed for 48 h in Davidson’s fixative and subsequently dehydrated through a series of ethanol and processed according to routine histological protocols.

For conventional histological examination, 3 μm thick sections were stained with haematoxylin & eosin (HE) and thoroughly screened for all developmental stages of the apicomplexans.

The ISH methodology, which was applied to 10 selected sections of each infected mollusc host species, roughly followed the procedure of Morris *et al*.^46^ and Holzer *et al*.^47^, with modifications which consisted of lower concentration of proteinase K. Histological sections, 7 µm thick, were hydrated and permeabilized with 10 µm/mL proteinase K in Tris-buffered saline (TBS) pH 8 for 12 minutes at 37 °C followed by a 2 × 5 min washing in PBS. Samples were then post-fixed in 0.4% paraformaldehyde in PBS for 15 min and subsequently washed for 2 × 5 min in distilled water. In order to prevent non-specific binding, sections were exposed to 10% hydrogen methanol (H_2_O_2_) for 10 min and then washed in distilled water for 2 × 5 min. Following that, the sections were dried in 45 °C for 10–12 min to be able to omit the time-consuming pre-hybridization step. Samples were enclosed with Frame-Seal^TM^ (Bio-Rad, Sundbyberg, Sweden) chambers and equilibrated in hybridization buffer consisting of 100 µg ml^−1^ calf-thymus DNA, 1.5 ng ml^−1^ of each of two 5′ biotin labelled oligonucleotide probes and 4x saline-sodium citrate buffer (SSC) in TBS containing 0.5% Ficoll, 0.5% polyvinylpyrrolidone, 0.5% bovine serum albumin. The following two probes were used: 790r 5′ ACACSCTTGAAGCACCCTAC 3′ and SC2-1370r 5′ TCCTTCATATGTCTGGCACTAG 3′ (5). The sections were sealed, denatured at 95 °C for 4 min followed by a 60 min hybridization at 45 °C. Hybridization was followed by non-stringent and stringent washes with 2x SSC and SSC with 0.1% Tween 20 at 42 °C, respectively. Signal detection was achieved using incubation with horseradish peroxidase-labelled streptavidin (Dako, Agilent Technologies, Glostrup, Denmark) for 20 min at room temperature followed by 3 × 5 min washing in PBS (pH 7.4) and visualized with a DAB Peroxidase Substrate (Vector Laboratories, Burlingame, USA). Haematoxylin was applied as a counterstain, after which sections were rapidly dehydrated in series of ethanol, transferred to xylol and mounted in resin based medium.

### Data availability

All data needed to replicate the study are within the paper and its Supplementary Information file.

## Electronic supplementary material

Fig. S1.
