# Supplementary material for: Harmless sea snail parasite causes mass mortalities in numerous commercial scallop populations in the northern hemisphere
Source: Sci Rep. 2018 May 18;8:7865. doi: 10.1038/s41598-018-26158-1 (PMC5959874; doi:10.1038/s41598-018-26158-1)
Supplement: Supplementary file 1 — Fig. S1. [file 41598_2018_26158_MOESM1_ESM.pdf]

Supplementary information

Harmless sea snail parasite causes mass mortalities in numerous commercial scallop populations in the northern hemisphere

Árni Kristmundsson*[1], Mark Andrew Freeman[2].

[1] Institute for Experimental Pathology at Keldur, University of Iceland, Fish disease Laboratory, Keldnavegur 3, IS-112 Reykjavík, Iceland.
[2] Ross University School of Veterinary Medicine, Basseterre, St. Kitts, West Indies.

*Corresponding author: Árni Kristmundsson, arnik@hi.is
Co-author: Mark Andrew Freeman, mafreeman@rossvet.edu.kn

Supplementary Information Fig. S1.

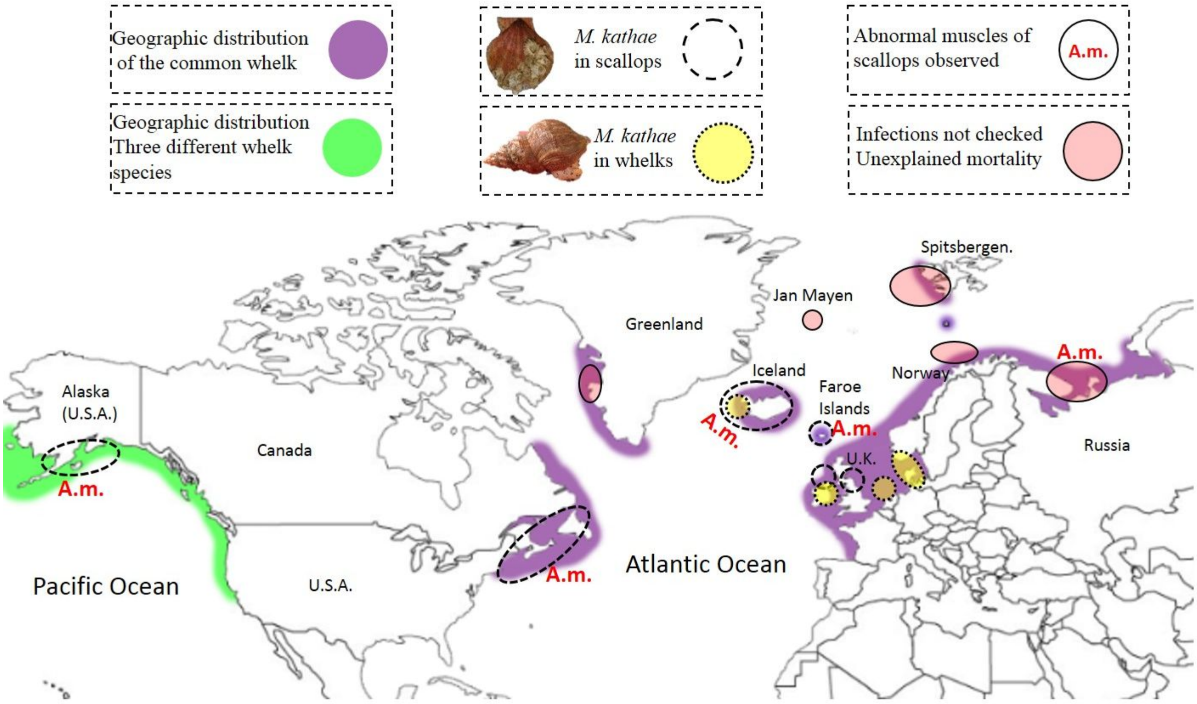

Fig. S1. Known distribution of *M. kathae* and whelks, and sites were abnormal condition of scallop populations have been experienced.

Known sites where abnormal mortality in scallops have been observed, clinical signs of disease similar to those caused by *M. kathae* and sites where the apicomplexan has been observed in scallops and whelks. Furthermore, it shows areas where unexplained mass mortalities of scallops have been observed and no examination with regard to infectious diseases performed. The shaded areas represents the geographical distribution of two different species of whelks, i.e. the common whelk, *Buccinum undatum*, and the sinuous whelk species, *B. plectrum*, a possible host for *M. kathae*.
